# Determination of Vibration Parameters of Deformable Structures Using Different Dynamic Loading Schemes and Types of Fiber-Optic Strain Sensors

**DOI:** 10.3390/s26196179

**Published:** 2026-09-29

**Authors:** Valerii Matveenko, Grigorii Serovaev, Elizaveta Galkina, Andrey Fedorov

**Affiliations:** Institute of Continuous Media Mechanics, Ural Branch of Russian Academy of Sciences, 614068 Perm, Russia; mvp@icmm.ru (V.M.); galkina.e@icmm.ru (E.G.); fedorov@icmm.ru (A.F.)

**Keywords:** strain measurement, point and distributed fiber-optic sensors, forced and free vibration, natural frequencies and mode shapes, logarithmic decrement, strain amplitude–frequency curves

## Abstract

This paper presents several approaches for determining the vibration parameters of thin-walled deformable structures under different dynamic loading regimes using two types of fiber-optic strain sensors. The parameters considered include vibration amplitudes and frequencies, logarithmic decrements and strain mode shapes. The results are presented for a carbon fiber-reinforced polymer plate with nonstandard geometry. The measurements were performed using single-point fiber-optic sensors based on fiber Bragg gratings and distributed fiber-optic sensors based on Rayleigh scattering. The considered dynamic regimes included steady-state forced vibration and free vibration. Free vibration was initiated either by terminating steady-state excitation at a resonant frequency or by releasing the specimen from an initial static deflection imposed at different points. The experiments yielded strain amplitude–frequency curves under steady-state forced vibration based on measurements obtained with point and distributed fiber-optic sensors. The capability of distributed sensors to provide a spatial representation of strain, particularly the strain mode shapes, was demonstrated. The paper also presents resonant frequencies and logarithmic decrements determined for different types of dynamic response of the specimen.

## 1. Introduction

The relatively low bending and torsional stiffness of thin-walled structures result in their significant deformation response to the applied dynamic loads. The dynamic behavior of such structures has remained a focus of research for many decades, while advances in numerical and experimental methods continue to create opportunities for obtaining new results. One such opportunity is the application of fiber-optic sensors (FOSs) to analyze the dynamic behavior of structures. Over the past 20–30 years, these sensors have demonstrated several advantages over other sensor types. The small size of FOSs enables their embedding in materials and ensures that adding a sensor to relatively small test objects does not alter their dynamic characteristics. The simultaneous use of point and distributed FOSs combines the advantages of both sensor types, while distributed sensors are especially effective for measuring spatial strain distributions. Immunity to electromagnetic interference and lack of a power-supply requirement broaden the range of practical applications for FOSs.

The dynamic behavior of a structure can be characterized by its strain and stress under load, natural frequencies and mode shapes, frequency–response curves under steady-state forced vibration, and time-dependent strain response to dynamic excitation. The capabilities of FOSs for recording and analyzing these quantities have been reported in numerous publications. The sources cited in this section highlight the key aspects of previous research.

Most studies focus on determining natural frequencies and mode shapes. Results are reported primarily for beams, plates, and shell structures [1,2,3,4]. The ability to record changes in natural frequencies in real time should be noted [5,6,7,8]. Such changes are among the indicators of damage or degradation of the mechanical properties of materials. For example, modal analysis of a reinforced concrete column subjected to loads simulating seismic action showed that its first natural frequency decreased by more than a factor of two over time [5]. Studies [9,10] demonstrate the measurement of dynamic characteristics using sensors attached to or embedded in polymer composite materials. The ability to obtain information on a spatially continuous strain distribution using distributed FOSs is shown in [11]. That study demonstrates the simultaneous recording of bending and torsional modes in thin-walled structures, which are governed by different components of the displacement vector. In [12], an approach was proposed for reconstructing the mode shapes of a composite plate based on distributed FOS measurements and the inverse finite element method (FEM) within a digital twin framework. The advantages of distributed FOSs over conventional point accelerometers were demonstrated in measurements of the vibration modes of a helicopter blade model [13]. In [4], conventional piezoelectric accelerometers and FOSs were used to measure the dynamic response of a composite component. The results showed that FOSs can accurately determine natural frequencies and mode shapes. In [14], using a cantilever plate as an example, it was demonstrated that the time histories of the Doppler shifts in the carrier frequencies measured by distributed FOSs based on optical frequency-domain reflectometry (OFDR) can be used to determine the slopes of the vibration mode shapes and subsequently reconstruct the displacement mode shapes by numerical integration.

The dynamic measurement range of a FOS system is governed largely by interrogation speed. This corresponds to the interrogator sampling rate for point fiber Bragg grating (FBG) sensors and the reflectometer sampling rate for distributed FOSs based on Rayleigh scattering. Given known elastic constants, measured strain can be used to estimate stress values. These capabilities are demonstrated in [15] for FBG measurements of stress-wave propagation during impact pile driving and for distributed FOS measurements used to determine steel and bond stresses in reinforced concrete elements under monotonic and cyclic loading [16]. It should be noted that the reviewed literature did not explicitly address experimental results involving measurements of all components of the strain tensor and, consequently, obtaining information on stresses in structures that cannot be described using one-dimensional models.

Fiber Bragg grating FOSs are used to record high-frequency acoustic and impact signals, free vibrations, and other phenomena [17,18,19,20]. The resulting strain-time histories make it possible to evaluate dynamic response characteristics, including vibration amplitudes and frequency spectra [9]. Although distributed sensors cover a narrower dynamic-load frequency range than point sensors, they can record a distributed response to impact loading [21]. In [22], a method was proposed to improve the temporal resolution of distributed FOS measurements based on Rayleigh scattering. The dynamic strain field is treated as a two-dimensional function of position along the fiber and time and is reconstructed using two-dimensional deconvolution and deep learning methods. This approach enables a higher effective measurement rate while maintaining high spatial resolution.

The logarithmic decrement is an important dynamic characteristic that can be difficult to determine accurately. Various studies have demonstrated that FOSs can be used to determine this characteristic [23,24], including the determination of logarithmic decrements at different frequencies [25] and their comparison with results obtained using accelerometers [26].

This study demonstrates different approaches to determining the principal vibration parameters of a carbon fiber-reinforced polymer (CFRP) plate with a wing-like geometry. The considered parameters (resonant frequencies and strain mode shapes, strain amplitude–frequency curves and logarithmic decrements) were determined by applying different dynamic loading schemes and measuring strain with two types of fiber-optic sensors: point sensors based on FBGs and distributed sensors based on Rayleigh scattering. A distinctive feature of this study is its comprehensive approach to determining the principal vibration parameters of structures by comparing different loading schemes and FOS types. The reported results can guide the selection of the most appropriate measurement strategy for a given thin-walled structural configuration and the available experimental capabilities. The key elements of the methodology for determining vibration parameters, demonstrated using a structurally representative laboratory specimen, can also be applied to actual thin-walled structures.

## 2. Experimental Description

**Figure 1:**
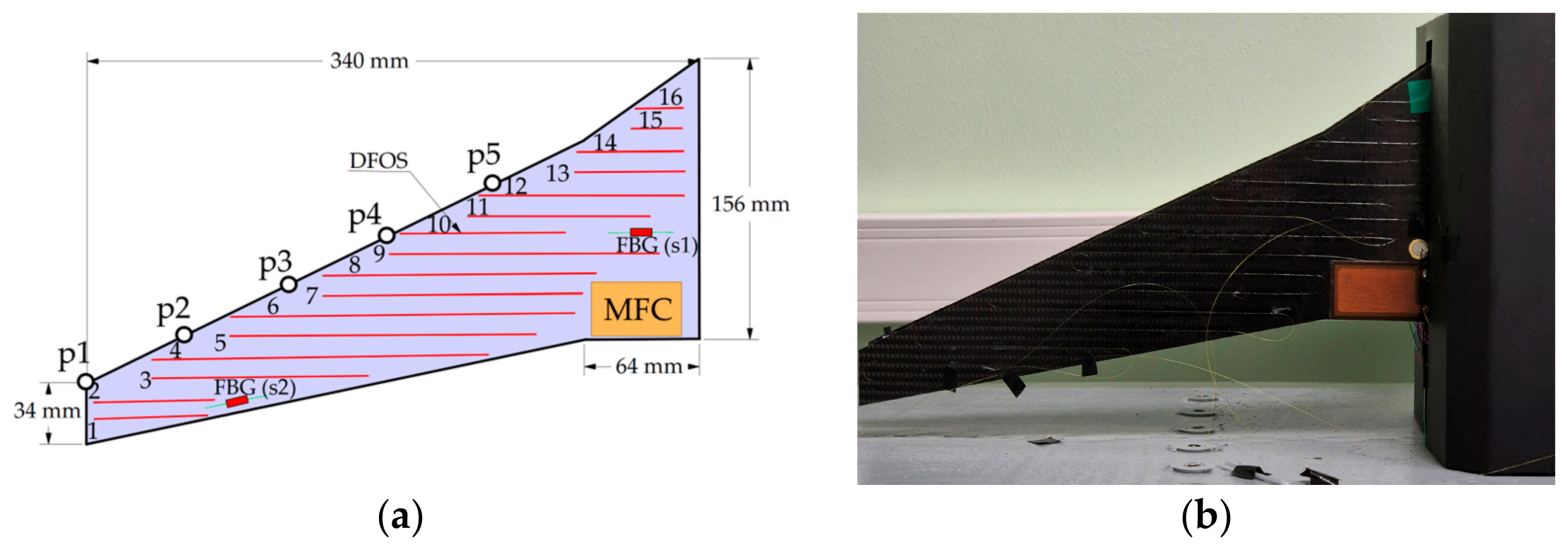
Schematic diagram (**a**) and photograph (**b**) of the specimen showing the locations of the point and distributed FOSs.

A wing-shaped plate rigidly clamped along one edge was used to investigate the dynamic response and determine the natural frequencies, strain mode shapes, resonant strain amplitudes, and logarithmic decrements (Figure 1). The plate was manufactured by compression molding from 15 prepreg plies, each 0.2 mm thick. The prepreg has a woven reinforcement architecture. The warp and weft yarns are oriented at +45° and −45°, respectively, relative to the global *x*-axis. The overall plate thickness was 3 mm. The effective elastic properties are as follows: *E*_1_ = *E*_2_ = 45 GPa, *E*_3_ = 4.5 GPa, *ν*_12_ = 0.04, *ν*_23_ = *ν*_13_ = 0.3, *G*_12_ = 3.3 GPa, *G*_23_ = *G*_13_ = 2.7 GPa. Here, the axes of the local material coordinate system 1 and 2 are oriented along the warp and weft directions, respectively, while the local axis 3 is directed through the plate thickness. A Macro Fiber Composite (MFC) piezoelectric actuator of the size 50 × 30 × 0.3 mm was bonded to the plate surface. The principal actuation direction of the MFC actuator was aligned with the global *x*-axis of the plate. The piezoelectric actuator was driven by a signal generator through an amplifier, enabling controlled dynamic excitation of the specimen over a wide frequency range. The drive voltage was 200 V.

The specimen’s dynamic responses were measured using point FOSs based on FBGs and distributed FOSs based on Rayleigh scattering and the OFDR method. FBG sensors measure axial strain through the strain-induced shift in the Bragg wavelength of the reflected spectrum. In distributed FOSs based on Rayleigh scattering, the sensing section of the optical fiber is divided into a sequence of local subregions, which can be regarded as a separate sensing element. Each subregion has a length corresponding to the gauge length, within which the spectral shift in the Rayleigh backscatter is determined. The magnitude of this shift is related to the change in axial strain along the longitudinal axis of the corresponding fiber segment. A 150 μm diameter optical fiber with a polyimide coating was used in the experiments. The FBG sensors had a grating length of 5 mm. The FBGs were inscribed in SM1500(9/125)P single-mode optical fiber using the phase-mask technique (Inversion Sensor Co., Ltd., Perm, Russia), after which the polyimide protective coating was restored over the grating regions. The FBGs had a Bragg wavelength of approximately 1550 nm and a spectral peak width of approximately 0.2 nm. A HYPERION si255 interrogator (Micron Optics, Inc., Atlanta, GA, USA) was used to acquire data from the FBGs. The spectral range of the interrogator configuration used was 1510–1590 nm, and the maximum data acquisition rate was 5000 Hz. According to the manufacturer’s technical documentation, the wavelength accuracy is 2 pm, the long-term stability is 3 pm, and the wavelength repeatability is 2 pm. Assuming the nominal strain sensitivity of a standard FBG at 1550 nm, approximately 1.2 pm/µε, the specified wavelength accuracy of 2 pm corresponds to a strain equivalent accuracy of approximately 1.7 µε.

The optical fiber was bonded to the specimen surface using a cyanoacrylate adhesive after the surface had been degreased. The estimated thickness of the adhesive layer is 0.05–0.1 mm. In [27], it was shown that, with this bonding configuration, the material strain in the measurement region corresponds to the FOS readings. The locations of the two FBG sensors (*s*1, *s*2) were selected based on a finite element analysis of the natural mode shapes. Based on this analysis, regions with the highest *ε_x_* strain were identified for the first two modes. The arrangement of the point and distributed FOSs on the specimen surface is shown in Figure 1.

According to the Nyquist criterion, the 5 kHz sampling rate of the FBG interrogator provides a theoretical upper frequency limit of 2.5 kHz for vibration measurements. Strain measurements with the distributed FOSs were performed using an OSI-D backscatter reflectometer (ETSC Technologies, Hong Kong, China) with a maximum sampling rate of 100 Hz.

Two vibration regimes were investigated: steady-state forced vibration at several excitation frequencies and free vibration initiated either by terminating resonant excitation or by releasing an imposed static deflection.

## 3. Results

### 3.1. Results for Steady-State Forced Vibrations

**Figure 2:**
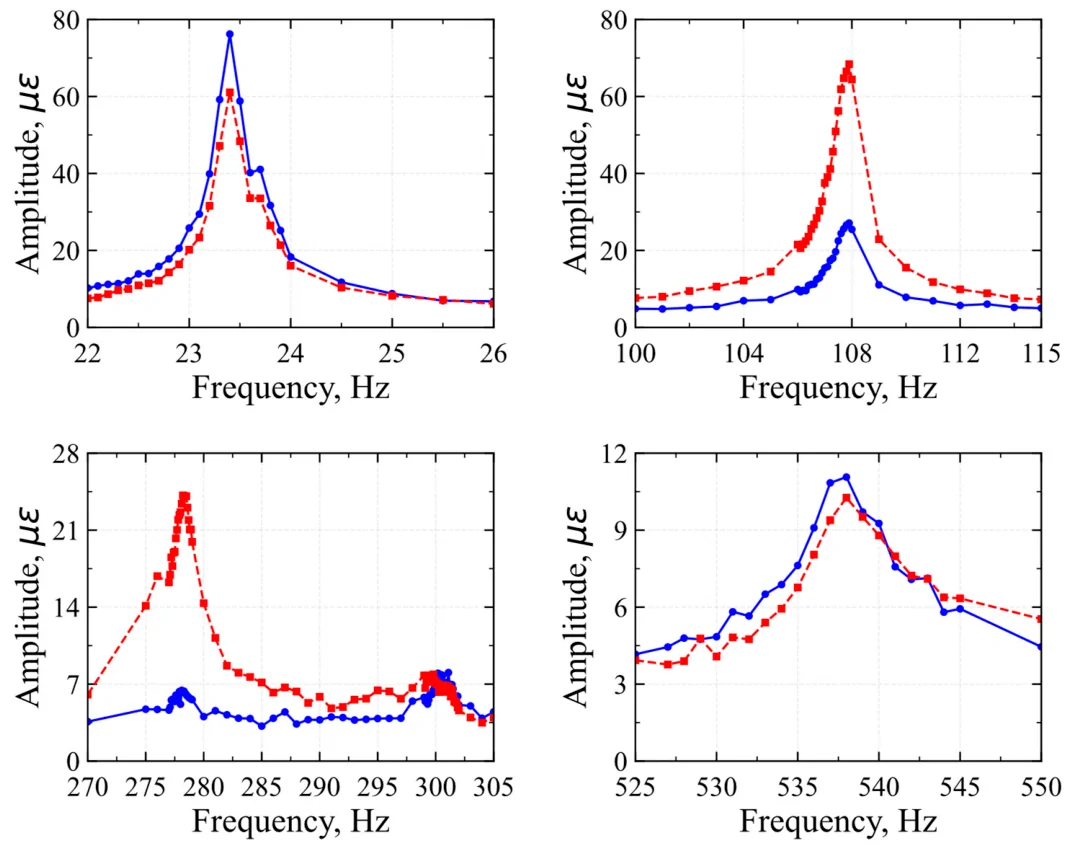
Frequency–response curves near the resonant frequencies (**—** s1, **—** s2).

Steady-state forced vibrations at different frequencies were generated using the piezoelectric actuator. During the experiments, the point FOSs measured peak strain amplitudes under harmonic excitation at frequencies up to 550 Hz. For the distributed FOSs, the measurement range was limited to 50 Hz by the capabilities of the reflectometer. Within the implemented frequency ranges, five resonant frequencies were identified using the point sensors and one using the distributed FOSs. Near the resonant frequencies, strain amplitudes were obtained by varying the frequency in increments of 0.1 Hz. Figure 2 shows the strain amplitudes measured by the point FOSs near the first five resonant frequencies. At each frequency, up to 50 measurement cycles were performed, and the mean amplitude was calculated.

**Figure 3:**
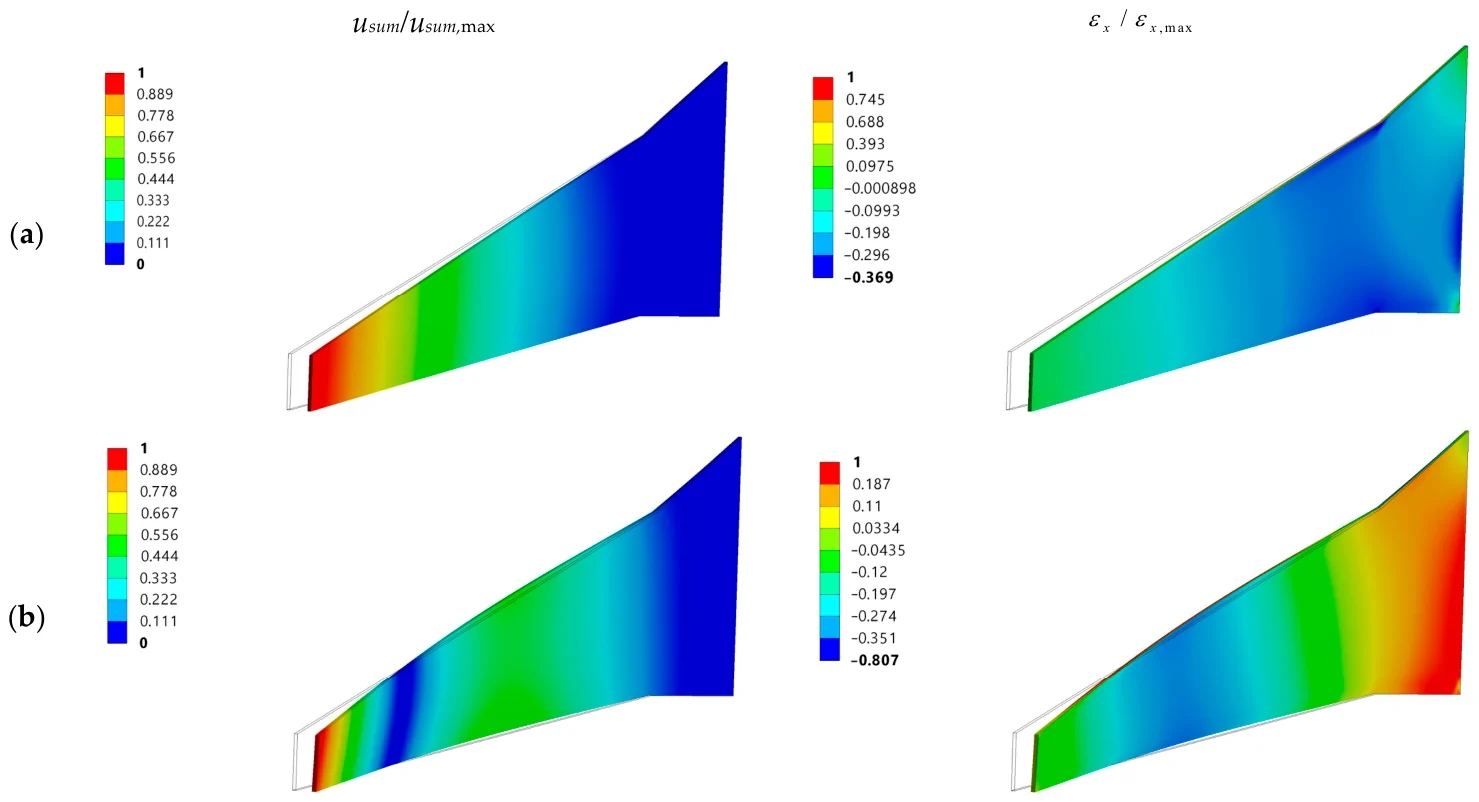

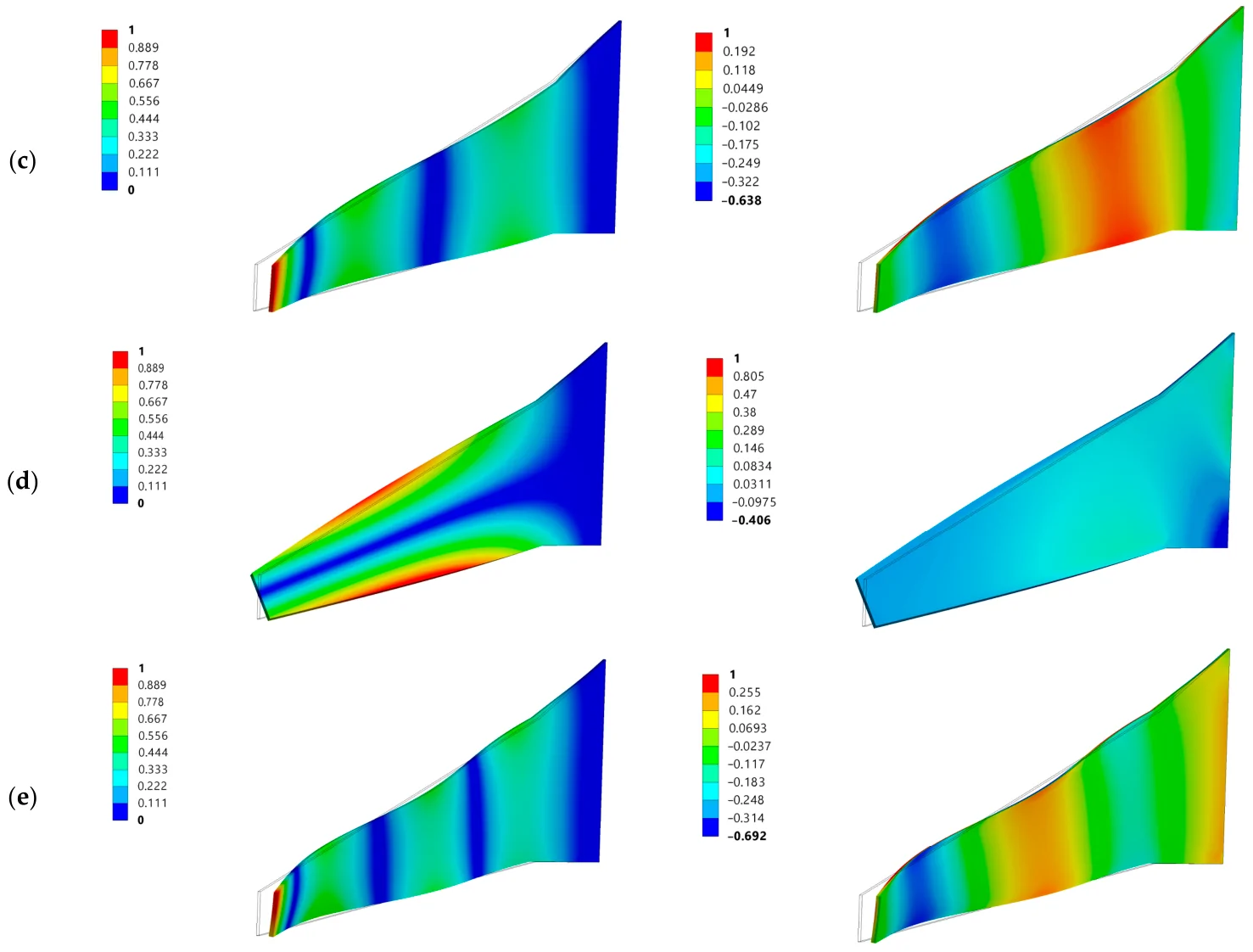
Numerically obtained mode shapes corresponding to the first five natural frequencies, represented by the normalized displacement magnitude *u_sum_*/*u_sum,_*_max_ and the normalized strain *ε_x_*/*ε_x_*_,max_; ((**a**–**e**) show the first five modes, respectively).

To assess the experimental results, a numerical modal analysis of the specimen was performed using an elastic material model. The analysis was carried out in ANSYS 2022 R1 using a three-dimensional finite element model. Numerical convergence was verified through an asymptotic convergence study with successive mesh refinement. Figure 3 presents the first five numerically obtained mode shapes in terms of the normalized total displacement magnitude *u_sum_/u_sum,_*_max_ and the normalized strain component *ε_x_*/*ε_x_*_,max_. Here, $u_{\mathrm{sum}}=\sqrt{u_{x}^{2}+u_{y}^{2}+u_{z}^{2}}$ Each field was normalized separately for each mode.

Table 1 presents the experimental resonant frequencies and the natural frequencies obtained numerically using an elastic material model. The quantitative difference between these values may reflect uncertainty in the material properties, boundary conditions and omission of damping in the natural frequency calculation. For most structural materials, this difference is small, and the comparison presented here is one means of demonstrating the reliability of the experimental results. Although the experimental and numerical results agree reasonably well, the absolute difference between them increases with resonant frequency. This appears to be associated with the change in the dissipative properties of the material as the vibration frequency increases, as is commonly observed for many materials. The numerical mode shapes explain the low strain amplitudes measured by FBG sensor s1 at 278.2 Hz and by both sensors at 301.1 Hz. For the corresponding modes, the sensors are located in low-strain regions.

**Figure 4:**
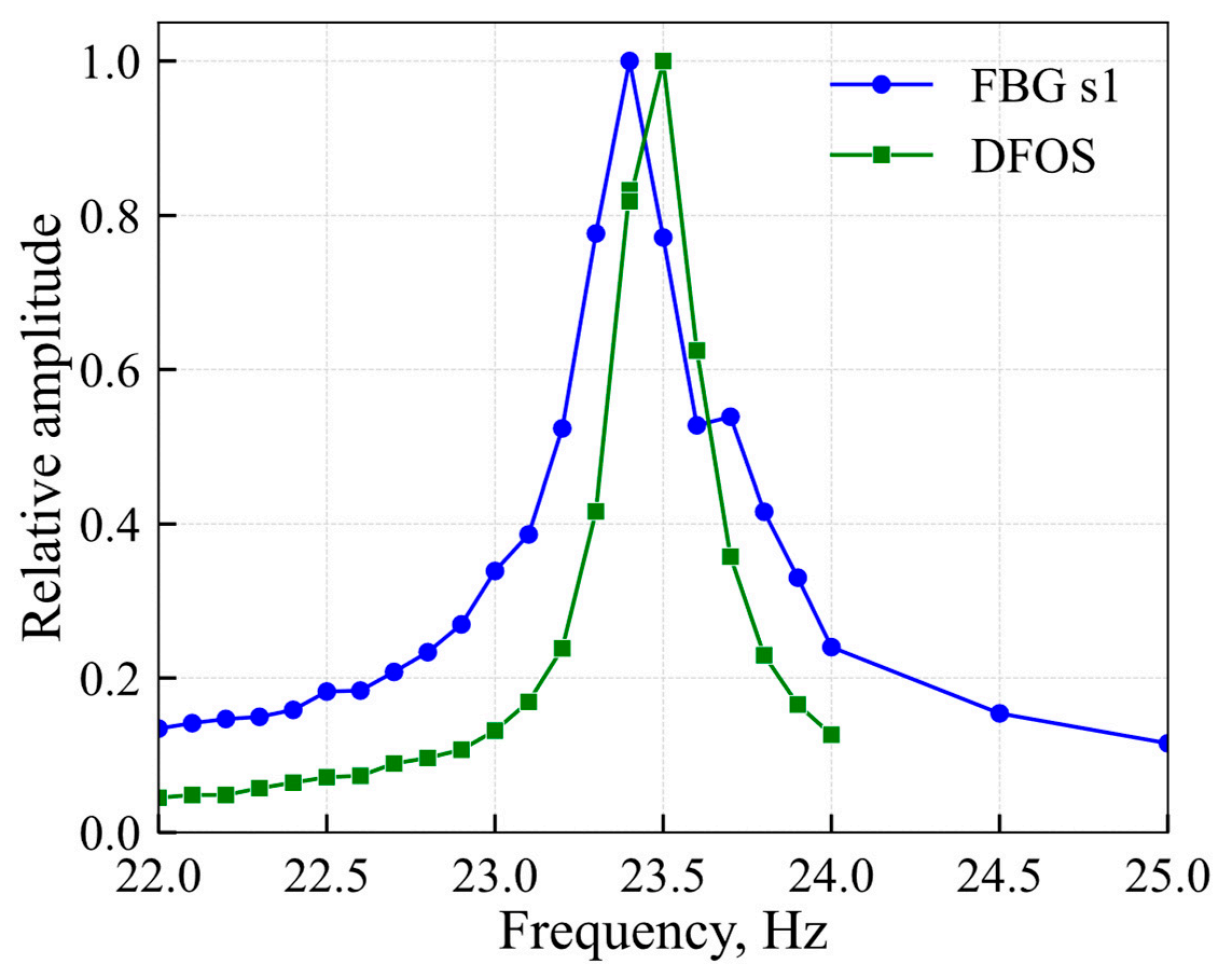
Relative vibration amplitude as a function of frequency for the point and distributed FOSs (DFOS) near the first resonant frequency.

Because of the sampling-rate limit of the dynamic optical backscatter reflectometer, the distributed sensor strain amplitude–frequency curve was obtained only near the first resonance. Figure 4 compares the point and distributed FOS responses in this range. Because the sensors occupied different regions, each curve was normalized by its own maximum amplitude. The corresponding resonant frequencies differ by the experimental frequency increment (0.1 Hz) used to construct the frequency–response curves.

**Figure 5:**
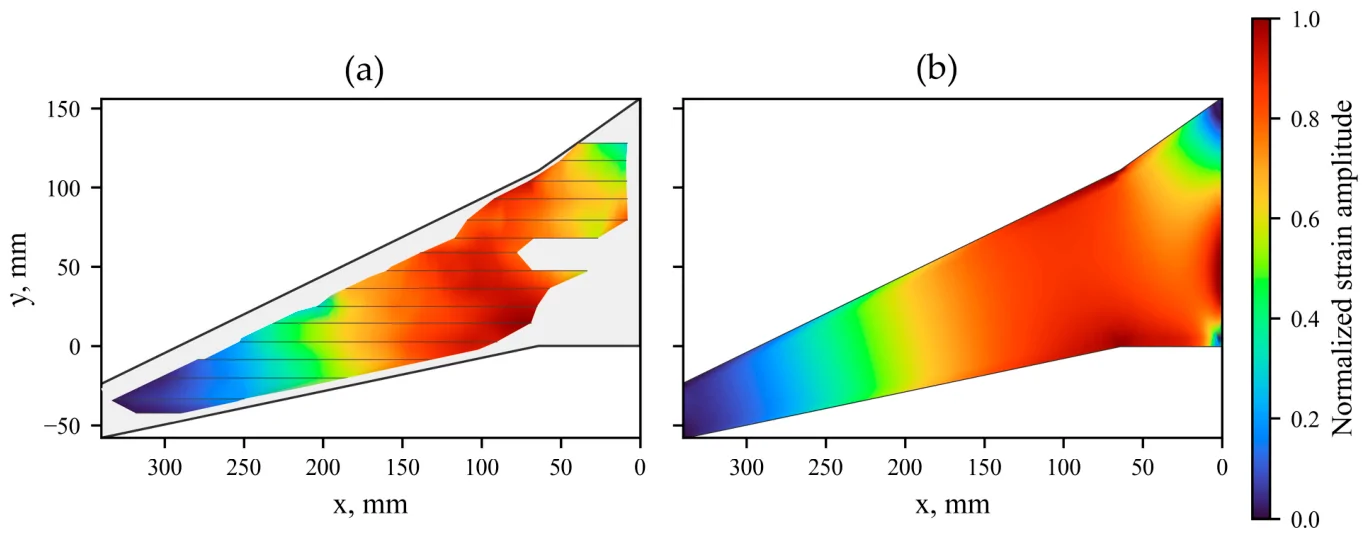
Normalized strain mode shape (*ε_x_*) at the first resonance frequency obtained from: (**a**) distributed FOS measurements; (**b**) finite element analysis.

Although distributed FOSs operate over a narrower measurement frequency range, they provide spatially resolved strain data. For the considered specimen, strain measured along the fiber path (Figure 1) was used to characterize the spatial strain distribution at the first resonance. The resulting strain distributions were normalized by the maximum strain values along measurement lines. The maximum difference between the experimental and numerical normalized strain values of *ε_x_* for the first vibration mode does not exceed 12%. The observed discrepancy may result from the idealization of the compression-molded CFRP as a homogeneous anisotropic material and from uncertainties in the elastic constants. The normalized strain mode shape represented by the *ε_x_* component of the strain tensor at the first resonance frequency was reconstructed from distributed FOS data within the measurement region using piecewise-linear interpolation over a triangular mesh generated from the coordinates of the measurement points. It is presented in Figure 5 together with the strain mode shape obtained from the finite element analysis. Because of the reflectometer’s sampling rate limitation, the present study could not demonstrate the ability of distributed FOSs to reconstruct modal strain distributions with steep spatial gradients. However, it was demonstrated in [28] that distributed FOSs can measure strongly nonuniform strain fields under static loading.

### 3.2. Results for Free Vibrations

Two cases of free vibration were considered. In the first case, these vibrations occurred after the actuator producing forced steady-state vibrations at the corresponding resonant frequencies was switched off. As a result, the specimen underwent free vibration in its natural modes, with the vibration amplitudes decaying over time. The resulting strain-time histories were used to calculate the logarithmic decrement *δ_i_* for the corresponding *i*-th vibration mode. It is defined as the natural logarithm of the ratio of two successive vibration amplitudes separated by one period. To incorporate more of the available experimental data and thereby improve the reliability of the logarithmic decrement estimate, the calculation was performed using pairs of peaks separated by *n* vibration periods, with multiple values of *n*.

$$\delta_{i}=\frac{1}{n}\ln\frac{A_{k}}{A_{k+n}},$$

where *A_k_* and *A_k_*_+*n*_ are the amplitudes of positive local maxima separated by *n* periods. For each excitation frequency investigated, several values of *n* were considered, where *n* denotes the number of periods separating the two peaks. For a fixed *n*, the logarithmic decrement was calculated for all admissible pairs of peaks in the analyzed signal segment, after which its mean value ${\bar{\delta }}_{i}$ was determined. The final value of the logarithmic decrement was estimated by analyzing the convergence of the mean value ${\bar{\delta }}_{i}$ as *n* increased.

For free-decay vibrations, the amplitude can be represented by an exponential function *A*(*t*) = *A*_0_*e*^−*αt*^, where α is the exponential decay coefficient. Taking the logarithm of this expression yields a linear form:

ln*A*(*t*) = ln*A*_0_ − *αt*.

The exponential decay coefficient *α* was determined by least-squares fitting of the experimental free-decay data. The resulting value of *α* was used to calculate the equivalent logarithmic decrement *δ_env_* = *αT*, where *T* is the mean period between the identified peaks. This quantity may be regarded as an alternative estimate of the logarithmic decrement, obtained not from the ratio of adjacent peak amplitudes but by approximating the envelope of the decaying signal.

**Figure 6:**
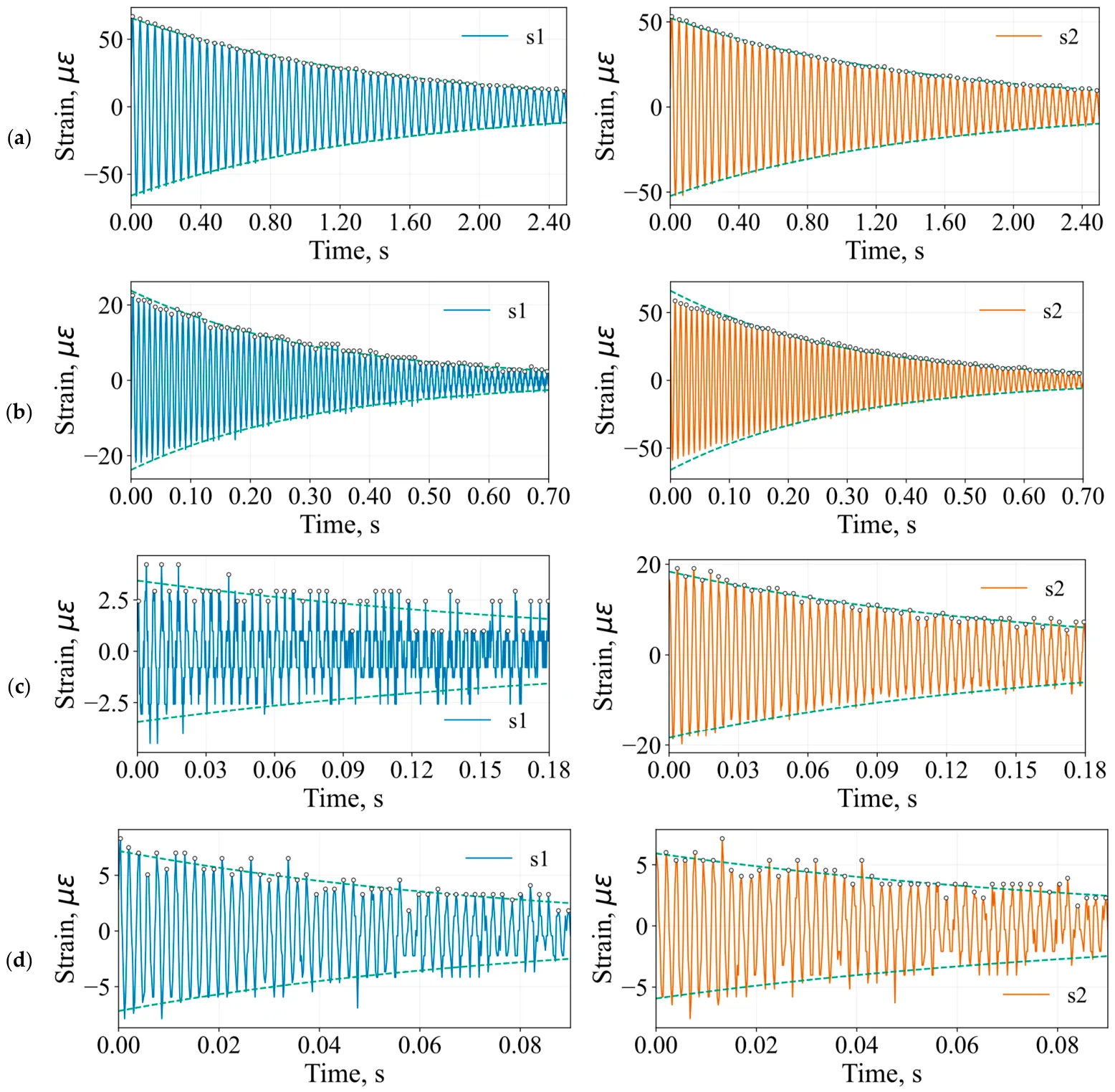
Free-decay strain-time histories at the investigated resonant frequencies, measured by FBG sensors s1 and s2 following steady-state excitation at the selected resonant frequencies: (**a**) 23.4 Hz; (**b**) 107.9 Hz; (**c**) 278.2 Hz; and (**d**) 538.0 Hz.

Figure 6 shows the experimental free-decay strain histories at the first, second, third, and fifth resonant frequencies obtained using FBG sensors s1 and s2. The green line in each plot is the envelope fitted to the positive local maxima, which are marked by points. Amplitudes of free-decay responses at the fourth resonant frequency (301.1 Hz) under the selected excitation regime were comparable with the sensor noise (approximately ±2 με). Consequently, the decay could not be determined reliably. It should also be noted that, for the same reason, the strain time histories recorded by sensor s1 at the third (278.2 Hz) resonant frequency do not exhibit a clear exponential decay.

These results indicate that free-decay strain-time histories used to determine the logarithmic decrement should have initial amplitudes exceeding the noise level of the measured strain signal by at least one order of magnitude. The logarithmic decrements calculated using the first (${\bar{\delta }}_{i}$) and second (*δ_env_*) approaches are presented in Table 2.

Depending on the vibration mode, the determined values of logarithmic decrements range from 0.018 to 0.030. The close agreement between the estimates obtained from the individual peak amplitudes and from the fitted decay envelope confirms the consistency of the two calculation approaches.

In the second free-vibration case, the plate was statically deflected by applying a load at each of five points (p1–p5) in separate tests. The load was then suddenly removed, and the plate underwent free-decay vibrations. Points p1–p5 are shown in Figure 1. The load magnitude was the same in all cases. The strain response was measured using point FBG sensors s1 and s2 at a sampling rate of 5 kHz.

**Figure 7:**
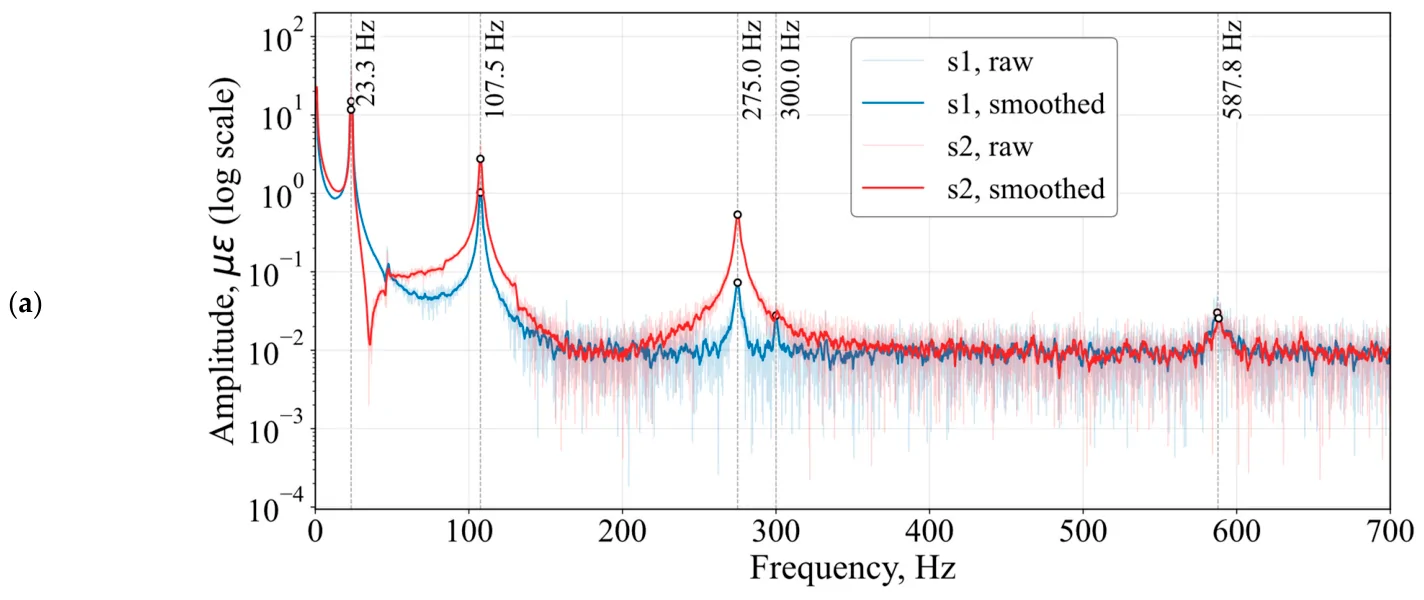

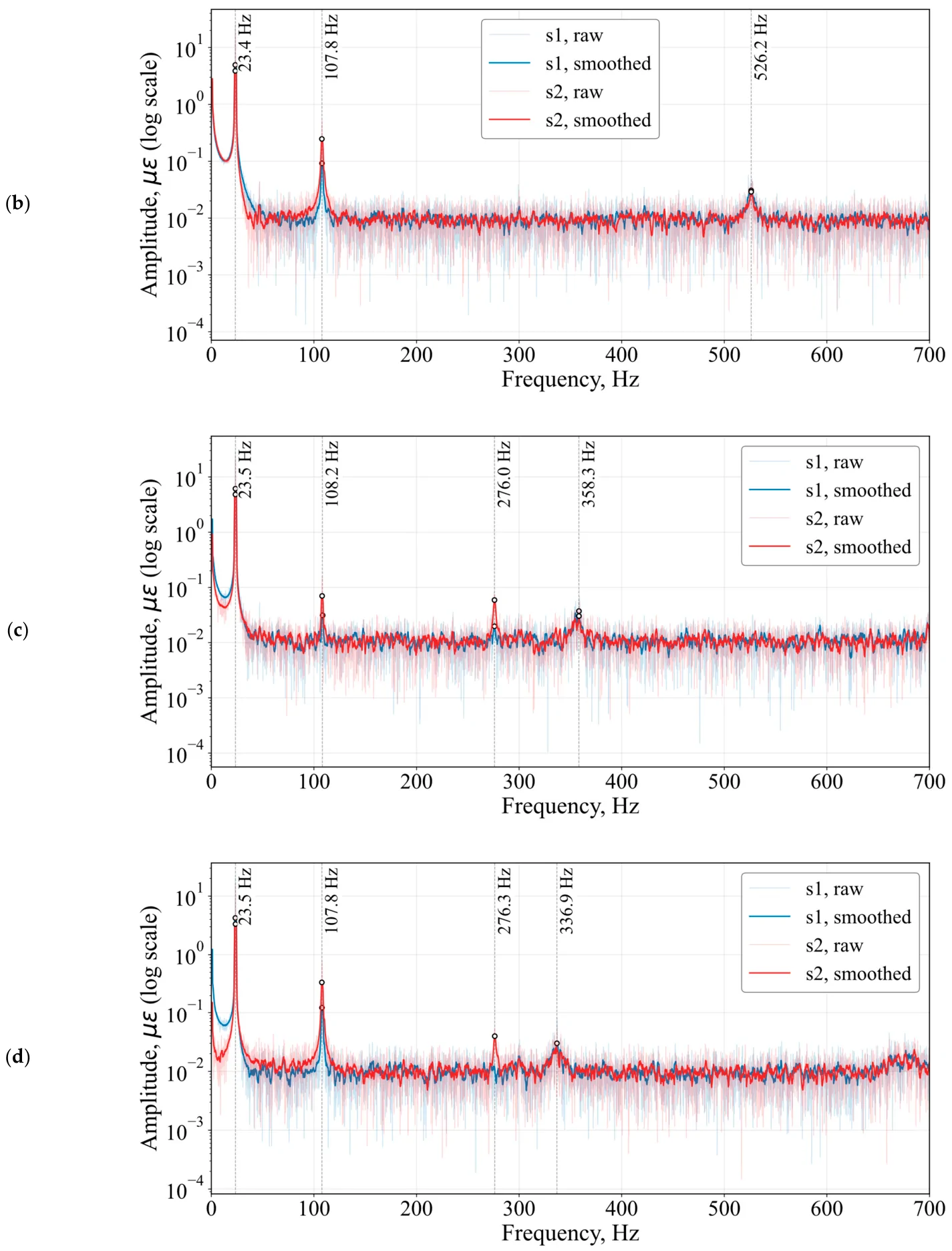

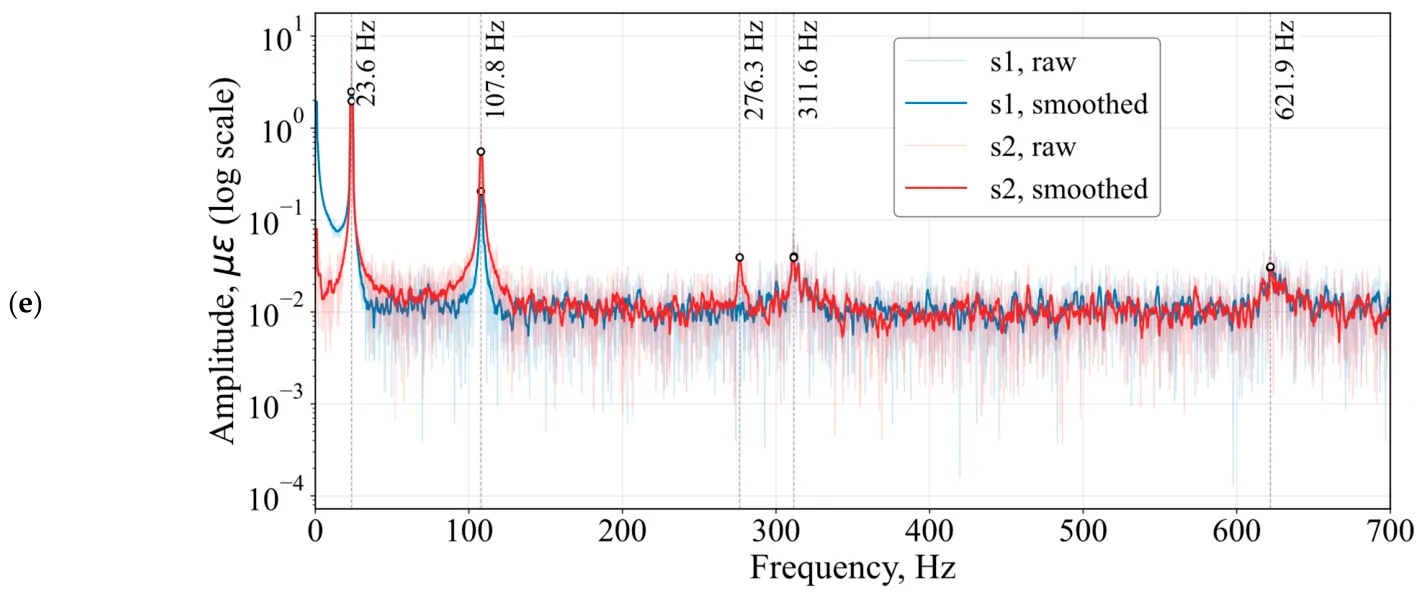
Spectral response plots of the specimen for initial vibration excitation at points (**a**) p1; (**b**) p2; (**c**) p3; (**d**) p4; and (**e**) p5.

Only the signal segments corresponding to free-decay vibrations were analyzed. For each segment, the constant component was removed from the strain-time history, after which the amplitude spectrum was calculated using a windowed Fourier transform. To identify the dominant frequency components, the spectrum was smoothed in the frequency domain, and peaks were detected over the range of 10–700 Hz. Figure 7 shows the spectral responses of the structure obtained in separate tests after the force applied at one of points p1–p5 to produce an initial static deflection was suddenly removed.

The resulting spectra show that the first two resonant frequencies of the structure are consistently identified for all considered initial deflection cases. In all experiments, pronounced peaks occur near 23–24 Hz and 107–108 Hz, in good agreement with the results obtained under forced steady-state vibration. The resonant frequency near 278.2 Hz does not appear in every experiment and is most evident in the data from sensor s2. This is consistent with the frequency response results for steady-state forced vibration, which indicated that sensor s1 had low sensitivity to this vibration mode. The spectral peaks associated with the fourth and fifth modes have low amplitudes and depend more strongly on the initial deflection point. Table 3 lists the resonant frequency ranges and the loading cases in which these frequencies were recorded.

Following release from an initial deflection, the recorded response is a superposition of several natural vibration modes. Therefore, the individual signal components corresponding to the resonant frequencies were separated before the damping parameters were calculated.

In the first stage, the dominant frequencies *f_i_* were identified from the amplitude spectrum of the free-decay response for each experiment and each FBG sensor. Then, for each identified frequency *f_i_*, the strain-time signal was band-pass filtered near that frequency. The specified interval *f_i_* ± Δ*f_i_* was used as the filter passband. Its width was selected based on the frequency separation from adjacent spectral components and the amplitude of the peak under consideration.

A free-decay segment in which the vibration amplitude decreased monotonically was selected from the filtered signal. The damping parameters were determined in the same manner as for the free-decay response following resonant excitation. Table 4 presents the logarithmic decrement values for the first three resonant frequencies. Parameter *δ_RE_* denotes the logarithmic decrement determined from the free-decay experiment following resonant excitation, whereas *δ_ID_* denotes the corresponding value obtained after release from an initial static deflection. Blank entries in Table 4 indicate cases in which the logarithmic decrement could not be determined reliably because of the low strain response amplitude at the corresponding sensor location.

The logarithmic decrements obtained using the two excitation methods are relatively close for the first and third vibration modes. The largest differences are observed for the second mode, for which the values obtained following release from an initial deflection are lower than those obtained following resonant excitation. The estimates also exhibit some dependence on the initial deflection point. These differences may result from variations in vibration amplitude and modal participation, as well as from amplitude-dependent energy dissipation mechanisms.

## 4. Conclusions

Several strain-based approaches were evaluated for determining the vibration parameters of a CFRP plate of nonstandard shape under steady-state forced vibration and free decay. Point and distributed FOS measurements yielded strain amplitude–frequency curves, from which the resonant frequencies were identified. These experimental frequencies were compared with the natural frequencies obtained by the finite element model. Distributed sensing also provided a normalized spatial strain amplitude distribution at the first resonance. Point FBG measurements were used to identify modal frequencies and estimate logarithmic decrements for two free-decay cases: after resonant excitation was terminated and after release from an initial static deflection. The results obtained under the different loading schemes were compared.

The results can be used in practical applications to select an appropriate strategy for determining the vibration parameters of thin-walled structures. Preliminary numerical modal analysis is useful for selecting the locations of point FBG sensors and identifying which resonant frequencies fall within the measurement bandwidth of the available reflectometer. Within this bandwidth, distributed FOSs can be advantageous because they provide spatially resolved measurements of axial fiber strain. Such measurements may support structural condition monitoring by revealing changes in free-vibration strain patterns associated with damage. Among the loading schemes considered, resonant harmonic excitation followed by switch-off provided the most direct determination of resonant frequencies and mode-specific logarithmic decrements. Release from an initial static deflection was simpler to implement but required additional processing to separate the modal components.

## Figures and Tables

**Table 1:** Experimental resonant frequencies and corresponding FEM natural frequencies.

Mode Number	Experimental Resonant Frequency, Hz	FEM Natural Frequency, Hz
1	23.4	23.7
2	107.9	105.7
3	278.2	270.7
4	301.1	294.3
5	538.0	527.4

**Table 2:** Logarithmic decrements determined from experimental free-decay strain data following excitation at the corresponding resonant frequencies.

	*f* = 23.4 Hz		*f* = 107.9 Hz		*f* = 278.2 Hz		*f* = 538 Hz	
	s1	s2	s1	s2	s1	s2	s1	s2
${\bar{\delta }}_{i}$	0.029	0.029	0.028	0.030	0.019	0.022	0.022	0.018
*δ_env_*	0.029	0.029	0.028	0.030	0.019	0.022	0.022	0.018

**Table 3:** Comparison of resonant frequencies identified from steady-state frequency–response curves and free-decay spectra following release from initial deflections.

Resonant Frequency Under Steady-State Harmonic Excitation, Hz	Modal Frequency Range Identified from Free-Decay Spectra, Hz	Initial Deflection Points
23.4	23.3–23.7	p1–p5
107.9	107.5–108.5	p1–p5
278.2	275.0–276.3	p1, p3, p4, p5
301.1	300.0–311.7	p1, p5
538.0	490.7–588.2	p1, p2

**Table 4:** Logarithmic decrements determined from FBG strain responses during free decay following resonant excitation and following release from initial deflections imposed at points p1–p5.

Mode	FBG Sensor	*δ_RE_*	*δ_ID_*				
			p1	p2	p3	p4	p5
Mode 1	s1	0.029	0.027	0.026	0.026	0.025	0.026
	s2	0.029	0.026	0.026	0.026	0.025	0.025
Mode 2	s1	0.028	0.022	0.021	0.024	0.020	0.019
	s2	0.030	0.022	0.022	0.024	0.020	0.020
Mode 3	s1	0.019	0.022	-	0.023	-	-
	s2	0.022	0.022	-	0.021	0.018	0.017

## Data Availability

The raw data supporting the conclusions of this article will be made available by the authors on request.
